# Safety of liver resection and effect on quality of life in patients with benign hepatic disease: Single center experience

**DOI:** 10.1186/1471-2482-11-16

**Published:** 2011-07-26

**Authors:** Carsten Kamphues, Sabine Engel, Timm Denecke, Roberta Bova, Michael Hippler-Benscheidt, Gero Puhl, Peter Neuhaus, Daniel Seehofer

**Affiliations:** 1 Department of General, Visceral and Transplantation Surgery, Universitätsklinikum Charité, Campus Virchow Klinikum, Humboldt-Universität, D-13353 Berlin, Germany; 2 Department of Radiology, Universitätsklinikum Charité, Campus Virchow Klinikum, Humboldt-Universität, D-13353 Berlin, Germany

## Abstract

**Background:**

Although liver resection has long been established for selected patients with benign hepatic disease, the success of surgical treatment of these patients cannot be evaluated exclusively through postoperative morbidity and mortality. Therefore, the aim of the study was to prove the safety of liver resection in the treatment of benign liver tumors and to evaluate the effect of surgical treatment on the patients' qauality of life.

**Methods:**

A total of 146 patients who underwent liver resection because of benign liver tumors were included in this study. Postoperative outcome was assessed and patients evaluated their quality of life before surgery and at the present time using the European Organization for Research and Treatment of Cancer Quality of Life Questionnaire Core-30 (QLQ C-30).

**Results:**

The rate of serious (> grade 2) complications was 4.1% with no postoperative death. The quality of life assessment revealed an overall improvement of general health status after resection (0.7 vs. 0.56, p < 0.001) and additionally a significant reduction of 6 out of 9 symptoms. Furthermore, compelling benefits in the patients' social and emotional coping could be detected after surgery.

**Conclusions:**

Liver resection for benign liver disease is a safe procedure and leads to a significant improvement of quality of life in selected patients.

## Background

In recent years benign liver tumors have been diagnosed in larger numbers due to advantages in diagnostic imaging modalities [1-4]. Most common benign entities are hemangioma, followed by focal nodular hyperplasia (FNH) and adenoma, which together represent more than 80% of all benign liver tumors [3-5]. Rare benign liver diseases include for instance parasitic liver cysts, cystadenoma, caroli's disease and angiomyolipoma. Although data are heterogenous incidence of simple liver cysts is estimated to be approximately 5% [3-5]. Despite widespread availability of imaging modalities the differentiation between benign and malignant liver tumors remains a diagnostic challenge. While the accurate distinction between FNH and adenoma, as well as hepatocellular carcinoma, may be difficult, it is essential with regard to the appropriate treatment strategy [6]. Percutaneous liver biopsy often has limited benefit and must be regarded as contraindicated, especially in hypervascular tumors such as hemangioma [7].

Most benign tumors do not require aggressive treatment and the indication for resection of benign hepatic lesions remains controversial [3,4,8,9]. Today, hepatectomy is an accepted treatment for primary and secondary liver malignancies with low mortality-rates and morbidity of less than 30% [3,4,10,11], but major liver surgery for benign liver disease remains under discussion. Currently accepted indications for surgical resection of a benign liver tumor include suspicion of malignancy and severe or progressive tumor-related symptoms [3,4,11]. Large or growing hepatic adenomas should also be resected based on the risk of malignant degeneration and severe bleeding. In patients with parasitic liver cysts (e.g. echinococcus) a resection of the cyst is also recommended.

Postoperative morbidity and mortality as well as recurrence rate are the main factors evaluating the clinical outcome of patients undergoing major liver resection. However, a number of biological, social and clinical parameters that are meaningful for the patient are not reflected by these data, especially after resection of benign liver tumors. Based on the modern concepts of health-related quality of life (QoL) several questionnaires, such as the EORTC-QLQ-C30, have been developed in recent years, measuring the subjective outcome for patients after hepatobiliary surgery [12-15]. Assessment of quality of life, such as disease-free or overall survival, has become an important tool for the assessment of cancer patients [16,17], but no study has so far evaluated the quality of life liver resection has on patients with benign liver tumors.

The aim of the study was therefore to evaluate the postoperative outcome of patients who underwent liver resection due to benign liver disease in a primary hepatobiliary center. Furthermore, this study represents the first evaluation of quality of life after resection of benign hepatic tumors overall.

## Methods

Between April 2002 and May 2008 all patients who underwent liver resection for benign liver disease at our center were included in the study. Patients were excluded in case of occurrence of any malignant disease during the follow-up period.

For all patients eligible for the study the following data were collected in a prospective database: demographics, indication, details of surgical procedure, postoperative complications according to the score by Dindo et al. [18], follow up, including recurrence rate and survival, as well as quality of life before surgery and at the present time.

Indications for surgery were suspicion of malignancy, severe or progressive symptoms, diagnosis of parasitic liver cysts as well as diagnosis or suspicion of adenoma. Surgical procedures for benign liver tumors included: left and right hemihepatectomy, extended left and right hemihepatectomy, unisegmentectomy, plurisegmentectomy, atypical resection and combinations of these techniques. Patients undergoing laparoscopic fenestrations of liver cysts were excluded from the present analysis; however, patients who underwent formal liver resection for liver cysts or cystic liver tumors were included.

### Quality of life

Quality of life before and after surgery was assessed by sending a quality of life questionnaire to all patients included in this study after recovery from surgery. The patients were asked to evaluate their quality of life before surgery and at present time. Since there is no specific questionnaire for benign liver tumors available, the European Organization for Research and Treatment of Cancer Quality of Life Questionnaire Core-30 (QLQ C-30, Version 3.0) was used. This questionnaire is normally used in clinical cancer trials, but also adequate for assessing quality of life in patients with benign liver tumors, whose symptoms and discomfort are often similar to cancer patients. The questionnaire consists of 30 items incorporating 5 functional scales (physical, role, social, emotional, cognitive), 9 symptom scales (fatigue, nausea/vomiting, pain, dyspnoea, insomnia, loss of appetite, constipation, diarrhea, financial difficulties) and a global health scale [19]. Beyond the questionnaire all patients were asked if they would undergo liver resection for a benign hepatic lesion again, based on their present quality of life.

### Ethics

For this study ethical approval was obtained from the institutional ethical committee (EA2/047/09, Ethical committee of the Charité, University Clinic, Berlin) and all patients gave informed consent prior to the enrolment in the study.

### Statistics

Statistical analysis was performed using PASW Statistics 18.0 (SPSS Inc., Chicago, IL) and the R statistical software (Version 2.8.1, GNU/Linux). All quantitative data were expressed as median and range, unless otherwise indicated. Differences between quality of life parameters were tested using the Wilcoxon-test for paired samples. A p-value of less than 0.05 was regarded as statistically significant.

## Results

The study population consisted of 146 patients who had undergone liver resection due to benign liver disease at our center. There was an expected majority of female patients in the study population and preoperative blood samples did not reveal limited liver function in any of the patients. Relevant demographic and preoperative parameters of the entire cohort as well as the quality of life subpopulation are summarized in Table 1.

**Table 1:** Demographics and preoperative liver values of all patients and the Quality of Life subpopulation.

	All patients (n = 146)	QoL group (n = 81)
Age [years]	44 (13 - 74)	46 (16-74)
Gender ratio (male : female)	37 (25.3%) : 109 (74.7%)	16 (19.8%) : 65 (80.2%)
Tumor size [mm]	65 (10 - 230)	70 (8 - 230)
Preoperative bilirubine level [mg/dl]	0.5 (0.2 - 6.2)	0.5 (0.2 - 3.0)
Preoperative AST [U/l]	24 (6 - 259)	24 (8 - 259)
Preoperative ALT [U/l]	26 (4 - 775)	25 (5 - 775)
Preoperative Quick value [%]	97 (27 - 130)	98 (27 - 130)

Values are median (range).

### Diagnosis and indication

Main diagnosis in the study population was FNH in 45 patients (30.8%), followed by hemangioma in 28 patients (19.2%) and echinococcus cyst in 21 patients (14.4%). Table 2 categorizes the diagnoses for liver resection in this study.

**Table 2:** Diagnosis and type of liver resection of the entire cohort and the Quality of Life (QoL) subpopulation.

		All patients (n = 146)	QoL group (n = 81)
Diagnosis			
	FNH	45 (30.8%)	27 (33.3%)
	Hemangioma	28 (19.1%)	14 (17.3%)
	Adenoma	9 (6.2%)	6 (7.4%)
	liver cyst	15 (10.3%)	9 (11.1%)
	echinococcus cyst	21 (14.4%)	9 (11.1%)
	caroli's disease	13 (8.9%)	7 (8.6%)
	others*	15 (10.3%)	9 (11.1%)

Type of liver resection			
	hemi- or extended hemihepatectomy	77 (52.7%)	44 (54.3%)
	parenchyma-sparing resection	69 (47.3%)	37 (45.7%)

others*: liver abscess (6 patients), cystadenoma (5 patients), angiomyolipoma (4 patients)

The leading indications for liver resection were severe or progressive symptoms in 77 patients (52.7%), 48 patients (32.9%) underwent liver resection because malignancy could not be completely ruled out through preoperative measures. The remaining 21 patients (14.4%) were resected because of echinococcus cysts.

In 115 patients (78.8%) postoperative histopathological findings could confirm the most likely preoperative diagnosis, whereas in 31 patients (21.2%) the preoperative diagnosis turned out to be misleading.

### Operative procedures

The most frequently performed operative procedure was right hemihepatectomy in 39 cases (26.7%), followed by atypical resection (27 patients, 18.5%) and plurisegmentectomy (25 patients, 17.1%). Unisegmentectomy was performed in 17 patients (11.6%) whereas 16 patients (10.9%) underwent left hemihepatectomy. Extended hemihepatectomy was required in 15 patients (10.3%) on the right and in 7 patients (4.8%) on the left side.

The median operative time was 200 (77-517) minutes. In 32 patients (21.9%) a pringle maneuver was performed intraoperatively. 13 patients (8.8%) required blood transfusions during liver resection. The median stay on intensive care unit was 1 (0-10) day and patients were able to leave hospital after a median stay of 9 (2-55) days.

### Postoperative complications and follow up

There were a total of 25 postoperative complications (17.1%) and no postoperative death in the entire cohort. 10 patients had sterile fluid collections, of which 3 (2.0%) required percutaneous drainage. 2 patients (1.3%) suffered from postoperative bile leakage and required reoperation on the first postoperative day. Infectious complications (e.g. wound infection, cholangitis) occurred in 12 (8.6%) patients, while one patient suffered a middle cerebral artery stroke despite sufficient anticoagulation. This patient recovered well and was sent to a neurological rehabilitation 16 days after surgery. The rate of serious (≥ grade 3) complications was 4.1% (6 patients) in the study population.

During the follow-up period recurrent disease was observed in 13 out of 146 patients, representing a recurrence rate of 9% for the entire cohort. 6 out of these 13 patients developed liver cysts again, leading to a disease recurrence rate of 40% in this subpopulation, while 5 patients with FNH (11.1% of the FNH population) and one patient each with adenoma (11.1% of the adenoma population) and hemangioma (3.6% of the hemangioma population) had recurrence of their liver disease. However, none of these cases were recurrences in the common sense but patients rather developed growth of preexisting small lesions in the remnant liver based on the fact that all of these conditions can be of multifocal nature. After a median follow-up of 50 months (3-95 months) all patients are still alive.

### Quality of life

A total of 81 patients (55.5% of the entire cohort) who had returned a completed questionnaire were included in the quality of life assessment. Details on demographic data as well as diagnosis and type of liver resection are summarized in Table 1 and Table 2. The main indications for surgery were the occurrence of severe symptoms in 44 patients (50.6%) and suspicion of malignancy in 19 patients (19.8%). Postoperative morbidity rate was 17.2% (14 patients) and therefore in total not significantly different from the entire cohort. However, this subpopulation included 5 out of 6 patients with severe complications (6.2%). After a median follow-up of 56 months (17-76 months), 9 patients (11%) had recurrent disease, mainly after resection of benign liver cysts (4 patients = 40% of the liver cyst population). Likewise, all demographic as well as diagnostic and operative details of the quality of life subpopulation revealed no significant differences compared to the parameters of the entire cohort (data not shown). Therefore the quality of life assessment may be regarded as representative for the entire cohort.

### Quality of life assessment

Statistical analysis revealed a highly significant improved global health status after resection of benign liver tumor in our population (0.7 vs. 0.56, p < 0.001).

The Wilcoxon test for paired samples also showed a remarkable benefit in social (p = 0.03) and emotional functioning (p = 0.007) after surgery, whereas no impact on physical wellbeing and cognitive functioning was detected.

A reduction of symptoms after operative therapy of benign liver disease was observed in 6 out of 9 symptom scales. Especially for the symptoms pain (0.24 vs. 0.37, p = 0.001) and fatigue (0.3 vs. 0.41, p = 0.004) the postoperative improvement was highly significant. A detailed analysis of the EORTC QLQ C-30 questionnaire is summarized in Table 3.

**Table 3:** Results of EORTC QLQ C30 questionnaire before surgery and at present time.

	before Surgery		at present time		p
	**mean**	**SD**	**mean**	**SD**	

Global health status	0.56	0.30	0.70	0.22	**0.000**

Functional scales					
Physical functioning	0.83	0.22	0.85	0.17	0.16
Role functioning	0.73	0.34	0.77	0.31	0.23
Emotional functioning	0.57	0.29	0.66	0.26	**0.007**
Cognitive functioning	0.80	0.25	0.78	0.27	0.30
Social functioning	0.66	0.36	0.75	0.29	**0.03**

Symptom scales					
Fatigue	0.41	0.33	0.30	0.31	**0.004**
Nausea and vomiting	0.18	0.29	0.12	0.23	0.068
Pain	0.37	0.38	0.24	0.28	**0.001**
Dyspnoea	0.24	0.32	0.21	0.31	0.29
Insomnia	0.40	0.35	0.31	0.34	**0.027**
Appetite loss	0.19	0.32	0.11	0.23	**0.033**
Constipation	0.22	0.30	0.14	0.27	**0.027**
Diarrhoea	0.16	0.27	0.21	0.32	**0.039**
Financial difficulties	0.17	0.33	0.18	0.30	0.98

p< 0.05 is considered significant and in bold.

SD: Standard deviation

At the end of the follow-up period, 78 of 81 patients (96.3%) would again undergo liver resection due to benign liver disease from their present point of view.

## Discussion

Despite controversial discussion about the practice of liver surgery in benign liver disease, nowadays liver resection is well-established as treatment of selected patients. Despite ongoing advantages in imaging modalities in recent years, there still is a considerable proportion of patients with an incorrect preoperative diagnosis. In a study by Charny et al. only 42 out of 62 preoperative diagnoses (67.7%) could be confirmed postoperatively by histopathological examination [11]. In the present study the percentage of patients with correct preoperative diagnosis increased to 78.8%, largely due to improved preoperative diagnostics. However, in a relevant number of patients the differentiation between benign and malignant hepatic lesions remains challenging.

In the largest series in literature Feng et al. reported on 827 liver resections for benign hepatic tumors with a postoperative complication rate of 13.5%, showing that liver resection can be regarded as a safe procedure [20]. Similar results were published by Charny et al. [11], who reported on 155 patients undergoing liver resection due to benign hepatic disease with a postoperative morbidity of 21% and no postoperative death. These results can be emphasized by the present study showing an overall complication rate of 17.1% with a mortality of 0%. In this series a total of only 4.1% of the patients had serious complications (≥ grade 3) which required further intervention but all of them recovered well during the follow-up period; however the majority of postoperative complications can be regarded as minor. Comparable data have recently been published by Lordan et al. [21]. In a series of 79 liver resections the rate of serious complications was 1.3% and no postoperative death occurred. This leads to the conclusion that even major hepatic resections of benign liver tumors can be safely performed in specialized hepatobiliary centers. This fact might influence the decision-finding process in these patients.

Furthermore the present study underlines that the recurrence rate of benign liver disease after liver resection is low. In our study 9% of the patients had recurrent disease and none of them required re-intervention. This strengthens the hypothesis that according to the standard parameters, morbidity and tumor recurrence, hepatic resection reaches good results in the treatment of benign liver disease. However, there are some important aspects concerning the patients' subjective feelings, which are not reflected by these parameters. Different health-related quality of life tools like the EORTC QLQ-C30 or the short-form 36 (SF 36) have been developed during the last 15 years in order to measure the effect of different therapeutic strategies on patients life, mainly in oncological patients [22-24]. These days, quality of life tools have also been established in the evaluation of subjective outcome of common hepatobiliary procedures and are therefore incorporated into the decisions of clinicians and patients [12-14]. In a prospective longitudinal study Poon et al. showed a significant enhancement of quality of life in patients with hepatocellular carcinoma after hepatectomy [12]. Similar results were presented in another study by Martin et al. who revealed a significant improvement in long-term quality of life after major hepatectomy in patients with primary or metastatic liver cancer [13]. It is widely accepted that quality of life represents a parameter which is as important as disease-free survival or overall survival in cancer patients [15]. Nevertheless there is hardly any data about quality of life in patients with benign disease so far.

Although, particularly for those patients, for whom morbidity and mortality are not sufficient in evaluating the therapeutic success, this study is, to our knowledge, the first in literature that evaluates the impact of hepatic resection on the quality of life of patients with benign liver tumors. In several other studies it has been shown that 80-89% of all patients with preoperative complaints due to benign liver disease were asymptomatic after liver resection [8,11,21,25], but these studies only focused on a selection of all patients and often referred to a single symptom. In the present study it could be elucidated that hepatic resection leads to a significant improvement of global health status in the entire group of patients with benign liver disease. Beside a reduction of 6 out of 9 analyzed symptoms, a significant impact of liver resection on social and emotional functioning in these patients could be detected. The fact, that 96.3% of the patients would undergo liver resection again from their present point of view stresses the positive effect of liver surgery on patients' everyday lives. Although the interpretation of this study might be limited due to its retrospective character these results give a strong impression of the patients' subjective outcome after resection of benign liver tumors and underline the benefit of surgical treatment from the patients' point of view. The immanent bias of the return rate of the questionnaires in retrospective studies cannot be avoided in the present study as well. However, the Quality of Life subpopulation can be regarded as representative for the entire cohort and therefore all conclusions can be drawn for all the patients in this study.

## Conclusions

In conclusion, this study demonstrates that liver resection due to benign liver tumor can be performed safely in a specialized hepatobiliary center. Furthermore, this study is the first in literature to show that surgical treatment of benign liver lesions might lead to an improvement in quality of life by reducing symptoms and improving emotional and social functioning. Further prospective studies are crucial in order to support these results and thus might affect the decision-finding processes in patients with benign liver tumors.

## Competing interests

### Financial competing interests

• In the past five years have you received reimbursements, fees, funding, or salary from an organization that may in any way gain or lose financially from the publication of this manuscript, either now or in the future? Is such an organization financing this manuscript (including the article-processing charge)? If so, please specify. - No.

• Do you hold any stocks or shares in an organization that may in any way gain or lose financially from the publication of this manuscript, either now or in the future? If so, please specify. - No.

• Do you hold or are you currently applying for any patents relating to the content of the manuscript? Have you received reimbursements, fees, funding, or salary from an organization that holds or has applied for patents relating to the content of the manuscript? If so, please specify. - No.

• Do you have any other financial competing interests? If so, please specify. - No.

### Non-financial competing interests

• Are there any non-financial competing interests (political, personal, religious, ideological, academic, intellectual, commercial or any other) to declare in relation to this manuscript? If so, please specify. - No.

• The authors declare that they have no competing interests.

## Authors' contributions

CK had full access to all the data in the study and takes responsibility for the integrity of the data and accuracy of analysis. CK, SE, DS and PN were responsible for the study concept and design. The acquisition of data was performed by CK, SE, RB, TD and GP. CK, SE, TD, MHB, DS and GP were involved in the analysis and interpretation of data. The manuscript was written by CK, SE, RB, TD and MHB and critically revised by DS, GP and PN. All authors approved the final version of the manuscript.

## Pre-publication history

The pre-publication history for this paper can be accessed here:

http://www.biomedcentral.com/1471-2482/11/16/prepub
